# Factors that favor or hinder the acquisition of a digital culture in large organizations in Chile

**DOI:** 10.3389/fpsyg.2023.1153031

**Published:** 2023-03-09

**Authors:** Carolina Busco, Felipe González, Michelle Aránguiz

**Affiliations:** Faculty of Engineering and Sciences, Department of Industrial Engineering, Universidad Diego Portales, Santiago, Chile

**Keywords:** digital transformation, digital culture, digital leadership, digital strategy, Delphi method

## Abstract

Organizational culture is often perceived as a valuable strategic asset supporting business transformation and the exploitation of digital technologies. Still, it can also be the source of inertia that impedes change. The research question proposed is What factors favor or hinder the acquisition of digital culture in large organizations in Chile? The aim is to rank factors that promote a digital culture based on the perception of executives using the Delphi method. The expert panel was selected with strategic criteria, considering practical knowledge, up-to-date experience on the subject, and having high decision-making positions in large companies in Chile. The main statistics used are media, maximum, minimum, and average range, along with the search for consensus determined by the interquartile range and Kendall’s W concordance coefficient. Results show a high level of agreement on the importance of digital strategy and digital leadership factors when favoring a digital culture in large companies in Chile. However, large companies in Chile must pay attention to the conservative triad of elements that characterize Chilean work culture that considers the belief that changes are exclusively possible when commanded by the strategic apex, a hierarchical work culture that prevents collaborative work, and the rejection of disruptive change. These factors and cultural characteristics will likely hinder any attempt to succeed in a digital transformation plan.

## 1. Introduction

The rapid advancement of digital technologies in nearly all industries has changed the business environment, competitive dynamics, and customer demand (Lucas and Goh, 2009; Morakanyane et al., 2017; Westerman et al., 2019). While new technologies pressure digitalization even in areas that do not depend on them, digital technologies also present new opportunities for business growth. However, integrating and exploiting the opportunities that originate from digital technologies remains a significant challenge for companies. In order to maximize their benefits, the implementation of IT must be supported by an organizational transformation. Companies need to transform and digitalize all their business models and the existing organizational conditions, such as structures, processes, and culture (Clarke, 2014; Morakanyane et al., 2017). Leaders must recognize digital transformation as a strategic and fundamental paradigm shift that requires instilling a culture that supports change while enabling the overall company strategy (Hemerling et al., 2018).

Organizational culture is often perceived as a valuable strategic asset that has the potential to support business transformation and the exploitation of digital technologies (Westerman et al., 2019). Organizational culture can also be the source of inertia that impedes change (Lucas and Goh, 2009; Rodríguez, 2011). In research and practice, cultural change is perceived as essential for successful digital transformation in companies, especially when dealing with disruptive transformations brought about by new technologies (Westerman et al., 2019).

Digital culture in an organization is defined as a set of behaviors and habits to make the most of the potential of new technologies, aiming to transform the business model or organizational models to create value for customers, employees, and shareholders (Ochoa, 2016; Trushkina et al., 2020). Developing a digital culture is one of the critical pillars of digital transformation in companies. Those who implement it have a strong propensity to encourage risk-taking and innovation and develop collaborative work environments (Kane et al., 2017).

However, despite the perceived need for cultural change, most research only briefly addresses culture as a research topic. While unique values and generalized cultural attributes are sporadically proposed to foster successful digital transformation, there has not been a comprehensive analysis of which cultural values are crucial to the success of digital transformation. For this reason, this study intends to complement the wide range of factors and indicators associated with digital transformation in organizations that, despite having different approaches, may contain variables and dimensions in common.

The research question proposed is What factors favor or hinder the acquisition of digital culture in large organizations in Chile? The aim is to rank factors that promote a digital culture based on the perceptions of senior executives using the Delphi method. The Delphi method is one of the most used methodologies in scientific research today for problematic situations (Almenara and Moro, 2014), searching for consensus through the knowledge of a group of experts directly related to the objective, and topic of study (Aengenheyster et al., 2017). Results show a high level of agreement on the importance of digital strategy and digital leadership factors when favoring a digital culture in large companies in Chile. However, large companies in Chile must pay attention to the conservative triad of elements that characterize Chilean work culture, that considers the belief that changes are exclusively possible when commanded by the strategic apex, a hierarchical work culture that prevents collaborative work, and the rejection of disruptive change. These factors and cultural characteristics will likely hinder any attempt to succeed in a digital transformation plan.

## 2. Theory

This research proposes to investigate the concepts associated with digital transformation in organizations, understanding that they have a profound effect on society. These changes are understood from the theory of social systems in the context of an organizational society (Rodríguez, 2011). Thanks to industrialization, organizations are taking care of all the needs of society, reaching unprecedented diffusion, such as those that, through digital applications, solve the need to connect people to find companionship, thus transforming the social rules of seduction (Lipovetsky, 2020). Organizational systems, structurally coupled with their environment (Maturana and Varela, 1984) are affected by changes in society, while society is affected by the industrialization process and the effects generated by organizational systems. This co-evolution sustains constant increases in complexity in today’s society thanks to evolutionary acquisitions, which allows us to understand how “technique” generates profound effects on society and civilization (Luhmann, 2007).

The changes brought about by the digital economy are having a strong impact on organizations and the sciences that study them. Digital transformation is understood as the adoption of disruptive technologies to increase productivity, value creation and social well-being (Ebert and Henrique, 2018). It is also defined as the process used to restructure economies, institutions, and society at the system level through the incorporation of digital technology, triggering significant changes in their properties by strategically responding to their environment and optimizing their value creation processes (Galliers, and Jarvenpaa, 2010; Rachinger et al., 2018). In other words, with digitization information is digitized, with digitization the processes and roles that make up business operations are digitized, and with digital transformation the organization and its strategy are digitally transformed, affecting its culture.

Literature has shown great fertility when it comes to defining organizational culture, like a system of shared beliefs, norms and values, whether declared or practiced, that attribute meaning and an interpretative framework to the processes, behaviors and events that occur within an organization and that lead to the formulation of policies (Geertz, 1973). This system of shared values and beliefs interacts with the organizational structure, its members, and the control systems, producing norms of behavior (Harrison, 1972). It can also be described as a set of meanings and values that, as building blocks, configure the culture and are expressed through symbols, behaviors, and organizational structures (Garibaldi et al., 2009), allowing them to interpret such actions and judge them as appropriate or inappropriate. The evolution of a certain culture is attributed to an organizational learning process whether facing external or internal problems (Schein, 1990). The basis of these “institutional thoughts” is given by the culture that each organization has built (Douglas, 1996).

Schein’s (1990) may be the most influential definition and although important, does not consider that negative presumptions and beliefs are also part of the culture, and therefore have never served to adequately face subsistence problems or integration problems. Despite having a dysfunctional character, these cultural elements persist and can be used as guides for action (Rodríguez, 2011). Regarding these multiple interpretations of the concept of organizational culture, probably all the authors would agree in pointing out that they correspond to particular ways in which things are done within an organization. Therefore, organizational culture allows the reduction of complexity, by establishing a restricted framework of expectations (both individual and institutional) and possibilities of behavior, based on a general pattern of decision-making (Luhmann, 2010).

Digitization is the implementation and exploitation of digital opportunities using digital technologies. In the business context, this is associated with influencing the way processes are performed, changing business models, generating new revenue, and transforming how customers and businesses engage and interact (Bloomberg, 2018; Jovanović et al., 2018; Rachinger et al., 2018). This process of organizational transformation offers great competitiveness potential but requires adopting new operating patterns and innovative culture (Mirković et al., 2019). Thus, more technological companies have more significant profits and better-satisfied customers (Weill and Woerner, 2018).

The majority of existing models that analyze the digitization process in companies provide an incomplete picture of digital maturity, and the attributes that reflect a digital culture are not systematically integrated (Chanias and Hess, 2016; Remane et al., 2017; Teichert, 2019). However, many existing studies look at digital transformation using different indicators, therefore measuring various aspects of the phenomenon. For example, while some authors consider that a slight change enabled by technology (such as the implementation of a new ERP system) is an expression of digital transformation, others believe that this is a more radical and evolutionary process that takes place over time (Janowski, 2015; Loebbecke and Picot, 2015; Wang, 2016). Likewise, while some researchers associate digital transformation with business models and strategies, others see it as a paradigm or a process that must complete a series of stages (Berman, 2012; Berman and Marshall, 2014).

Digital transformation is a different concept. It is defined as the company’s ability to react and successfully use new digital technologies and procedures to drive significant change in its performance and business model (Cortellazzo et al., 2019). It represents applying digital capabilities to processes, products, and assets to improve efficiency, increase customer value, manage risk, and discover new monetization opportunities (Morakanyane et al., 2017). Similarly, Bertini (2016) points out that digital transformation affects individuals’ everyday experiences.

Digital transformation must be understood as a significant organizational change where innovation plays the primary role, affecting employees’ creative capacity (Villaplana and Stein, 2019). Therefore, it is effective when companies invest in developing digital skills and capabilities aligned with their corporate strategy. It must occur coordinately in all organizational dimensions: strategy, people and culture, structure and management systems, business processes, and technology (Ochoa, 2016). Consequently, digital maturity is a concept that reflects the adaptability of the organization to compete effectively in an increasingly digital environment. Therefore, it is a continuous process of adaptation to a digitally changing context, and as such, an organization must assess its digital maturity over time (Kane et al., 2017).

McKinsey & Company found that 80% of the companies surveyed started with a digital transformation. Still, only 14% reported an improvement in their performance, while only 3% indicated that the change had succeeded, confirming the challenge of digital transformation (Villaplana and Stein, 2019). Some of the most common organizational barriers to digital transformation are unclear vision and goal of digital transformation; lack of management understanding, knowledge, and experience; lack of leadership skills; lack of organizational agility; rewards and incentives that are not aligned with digital transformation; unclear measurement and reward system; lack of employee engagement; and employee resistance to change (Mirković et al., 2019).

Likewise, according to Kohnke (2017), the main barriers to digital transformation concerning organizational design are a lack of sense of urgency; unclear roles and responsibilities; unadjusted and rigid organizational culture; lack of internal talent for digital projects; inability to react quickly; failure to adopt an experimental and innovative culture; and inflexible business processes. In this context, organizational culture is increasingly seen as the main obstacle to digital transformation and effectiveness (Teichert, 2019). A culture conducive to digital transformation is a hallmark of maturing companies. These organizations have a strong propensity to encourage risk-taking, foster innovation, and develop collaborative work environments. Overcoming risk aversion is the most critical characteristic of digitally maturing cultures. They have conquered this cultural barrier by encouraging their organizations to experiment and accept the risk of failure (Kane et al., 2017).

Employees of digitally mature organizations describe their culture as more collaborative and innovative than other organizations and state that leadership has sufficient digital skills (Kane et al., 2015). Consistently research shows that around 80% of companies that focus on organizational culture consistently achieve high productivity results (Hemerling et al., 2018). Kanter (2001) proposes several characteristics of digital culture: continuously promote disruptive change, communicate fluidly inside and outside the organization, collaborate in the creation and delivery of value within the company and with third parties, share knowledge and create a shared identity, along with doing all the above quickly and agilely.

Overcoming risk aversion in digitally mature cultures and companies are primarily described by a culture of adaptability, meaning organizational learning, customer focus, and creating change. It is also relevant to a culture of ownership, which includes empowerment and team orientation. These cultural traits indicate higher levels of product and service innovation, creativity, and rapid response to changing customer and employee needs (Teichert, 2019).

In Chile, little research has focused on digital culture as a relevant dimension to boost digital transformation and maturity. A critical study was developed by the Santiago Chamber of Commerce, CORFO, and the PMG consulting firm, which presented the results of the fourth version of the Digital Transformation Index (ITD, 2021). These latest results detected an acceleration of digital transformation as an effect of the pandemic, where large companies increased the digitalization of their processes, especially those in the early stages. However, this increase in digital maturity was proportional to the level of maturity before the pandemic (ITD, 2021).

According to The Virtus Digital Maturity Index (IMDV), Chilean organizations’ digital maturity has evolved slowly despite the pandemic. Although there has been a significant leap in the incorporation of new technologies and processes, 67% of the largest companies in the country have the purpose of boosting a digital transformation. Only 52% have a clear and robust action plan to carry it out, explaining the quick 6.4% advance in digitalization compared to 2020. Therefore, the perception of high executives in large companies can help us identify factors that favor or hinder this adaptation process and recognize some blind spots of the system as a whole.

## 3. Methodology

Research design is empirical, non-probabilistic, cross-sectional, qualitative-quantitative, and exploratory. The research instrument uses the Delphi methodology, a systemic and interactive research tool that aims to obtain consensus through the compilation of specialized knowledge from a panel of independent experts on a specific and complex topic that would otherwise be difficult to study. The methodology uses questionnaires repeatedly sent individually, and results are returned in the form of feedback, creating a representative opinion of the group (Hallowell and Gambatese, 2010).

To guarantee results quality, four stages were conducted according to the research methodology: Formulation of the problem, selection of experts, elaboration, and launch of the questionnaires, and data analysis. High executives of large companies in Chile were considered the universe, where large companies are those composed of 200 or more workers (OCDE, 2010). The expert panel was selected with strategic criteria, given that a random selection is not acceptable for this methodology (Ludwig, 1997). The research problem conditions the expert’s profile and inclusion criteria (Needham and de Loë, 1990), considering practical knowledge, up-to-date experience on the subject, and having high decision-making positions in large companies in Chile, such as area managers, general managers, CEOs, CFOs, or board members. According to Mintzberg (1980), these positions are considered the strategic apex, responsible for deciding and implementing the company’s strategy. Other inclusion criteria were the variety of business sectors and willingness to participate (Ludwig, 1997; Hung et al., 2008).

Powell (2003) states that the number of experts may vary according to the research problem and available resources. Some panelists may drop out of the study due to other commitments or disinterest. Therefore, enough experts must be selected at the beginning of the process to ensure a qualified panel at the end of the study if some do not complete all rounds.

If the number of experts is too small, the information offered cannot be considered representative because the error decreases significantly for each expert added until it reaches seven. If the panel size exceeds 30, the prospecting improvement is minimal, so the cost increase does not compensate for that improvement (Astigarraga, 2003). Ludwig (1997) concludes that most Delphi studies use between 15 and 20 experts, while Landeta (1999) proposes between 7 and 30 participants.

**Figure 1:**
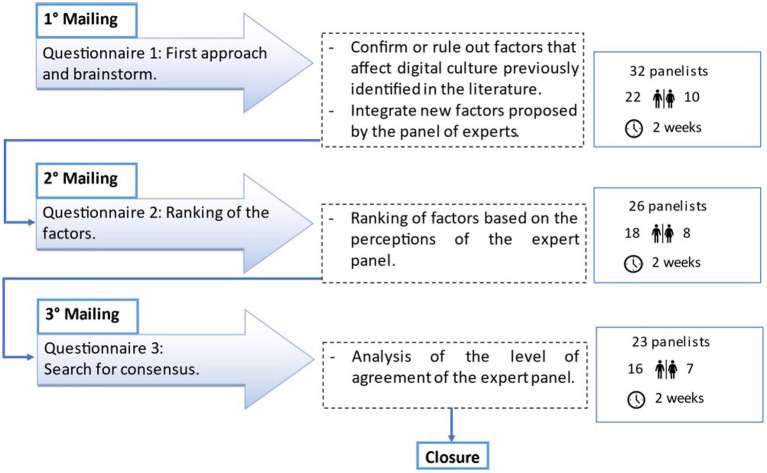
Data gathering process.

According to the criteria mentioned above, the process of expert’s selection began with an initial list of 50 possible candidates, to whom a formal proposal was sent by email with a brief description of the objectives, the expected number of rounds, and the estimated time of the complete process. Afterward, 32 experts responded with their willingness to participate and committed to the first round, 26 answered the second round, and 23 answered the third round of questionnaires. The data-gathering process is shown in Figure 1 and took place between April and May 2022.

An individual survey link was provided to the experts *via* email to ensure anonymity. An identifier code was assigned to each questionnaire to guarantee the confidentiality of the responses. The time laps between receiving and responding to each round were 2 weeks.

The sample included senior executives in the areas of financial services (3), services and it (4), food and beverages (2), telecommunications (3), logistics and supply chain (2), insurance (2), human resources services (2), health area (3), the energy sector (2 experts), construction sector (2), real estate sector (2), the automotive sector (1), the aeronautics and aviation sector (1), retail, textile and fashion industry (3). the positions held by the experts were general manager (3), area manager (13), deputy area manager (12), CEO (1), CIO (2), and director (1).

The initial questionnaire proposed a list of 14 factors from the literature, asking the panel of experts to indicate if each factor Does not contribute to the achievement of a digital culture, if it Hinders the achievement of a digital culture, or Favors it.

The second round added 12 new factors and two new dimensions (digital skills and technology) because of the experts’ proposals, increasing the total number of items in this questionnaire to 26. In this round, experts were asked to evaluate the importance of each factor associated with the acquisition of digital culture in large organizations in Chile, using a five-point Likert scale (1 = Not important, 2 = Somewhat significant, 3 = Moderately important, 4 = Important and 5 = Very important). The complete list of factors and their definitions grouped into five dimensions can be seen in Table 1.

**Table 1:** Complete list of factors and definitions grouped into dimensions.

Factors	Definition
**Digital culture**	
Customer orientation	Develop a rapid and efficient response capacity to customers’ changing needs (business models, processes, etc.).
Culture is open to change	Develop a capacity to adapt quickly to changes.
Tolerance for failure	Raise awareness within the organization that error is part of learning in the digital culture.
Experimental and innovative culture	Continuously experiment with digital technology and prototypes at low costs.
Risk tolerance	Encourage the organization to take risks.
Rewards and incentives aligned with digital transformation	Create reward systems to strengthen the position and development of highly qualified personnel (promotions, bonuses, awards, etc.).
Digital agility	Promote proactivity, adaptability, and a quick and adequate response to changes in the environment
Open communication	Develop virtual spaces to work collaboratively (e.g., JIRA, Teamwork Project Management, Microsoft SharePoint, etc.).
Integration of multifunctional teams	Encourage the generation of multifunctional/multidisciplinary teams to implement digital initiatives.
Disruptive change	Continuously promote disruptive changes within the organization.
Knowledge sharing	Create working groups uniting the digital generation and employees with experiences, generating intergenerational support.
Digital identity.	Generate a shared identity within the organization around digital.
Resistance to organizational change	Employee resistance to change, lack of participation, and commitment to supporting the company’s digital transformation.
Effective organizational communication	Generate spaces for inter-hierarchical communication related to digital transformation within the company.
**Digital Leadership**	
Digital leadership skills and abilities	Develop leadership (from top management) toward digital use of new technologies.
Digital leadership actions	Organizational leaders (senior management and directors) must build a supportive culture that encompasses collaboration, risk-taking, and experimentation.
**Digital strategy**	
Strategy alignment	Has a digital strategy aligned with the corporate strategy at a functional and operational level?
Clear vision and objectives of digital transformation.	Present a clear vision and objectives around the digital transformation both in the medium and long term.
Strategic business metrics to lead digital initiatives.	Successful digital initiatives require leaders to frame performance objectives around business goals defined by data rather than technical capabilities.
Organizational change management.	Properly manage the change, communicating the expectations and expected impacts.
Develop an area/team focused on organizational change management.	Develop a company change management area/team with a clear methodology.
**Digital skills**	
Training and digital education of employees	Permanently invest (at all levels of the company) in digital training of employees so that they develop up-to-date technological skills (e.g., Training)
Talent management.	Selectively invest in the most talented people and those most adaptable, curious, and flexible to develop, attract and retain the best talent.
Comprehensive development of digital skills and abilities.	Invest in the comprehensive development of digital skills and capabilities aligned with the company’s strategy.
**Technologies**	
Transformation and improvements of digital platforms (automation of core processes)	Integrate digital initiatives (Digitalization of information, processes, etc.) throughout the organization.
Digital skills.	Promote tools within the company without the need for very advanced programming knowledge.

In the last round, the experts were presented with three pieces of data: the eight factors that did not reach consensus in the second round, the score that the expert provided, and the average score of the responses considering all participants from the previous round. After reviewing the group statistics, each participant decided whether to change or keep their last answer. In addition, we included an open question for each item, asking why they chose to maintain or reconsider their previous score. This analysis allows for a measure of consensus and the convergence of opinions, reducing variation in the responses, and providing additional information on the interpretation and why the experts disagreed on the disputed factors (Heiko, 2012).

This research methodology was chosen because of its flexibility to adapt to many scientific disciplines. The method also uses controlled feedback that allows participants to reflect on a specific issue and participatory nature in constructing meaning among experts. In addition, it uses anonymity to avoid group think and the possibility of gathering qualitative and quantitative data to enrich the analysis process (Almenara and Moro, 2014; Reguant Álvarez and Torrado Fonseca, 2016; López Gómez, 2018). The Delphi method is widely used for organizational studies (Bhardwaj and Patnaik, 2019; García-Vidal et al., 2021; Kerpedzhiev et al., 2021; Vax et al., 2021) and project management research (Cafiso et al., 2013; Gajić and Palčič, 2019; Naji et al., 2022), including areas as different as civil engineering (Kermanshachi et al., 2020), health (Nasa et al., 2021), technology (Andersen, 2022), among many others.

Some recent work on organizational digital transformation explored which business process management capability areas will become relevant in view of digitalization (Kerpedzhiev et al., 2021). Using a Delphi study with international experts from industry and academia, this study updated business process management capability framework and identified challenges and opportunities. Related to organizational culture, García-Vidal et al. (2021) examined the relationships between organizational values and the performance indicators of an organization using a Delphi method. This study sampled two work teams and proved the relationship between values and customer satisfaction directly and productivity indirectly. Cafiso et al. (2013) used the Delphi method to evaluate opinions of public transport managers on bus safety. After a multi-round Delphi process, Kendall’s algorithm was used to evaluate the level of concordance, showing that the majority of the proposed items were considered to have great potential for improving bus safety.

## 4. Results

**Figure 2:**
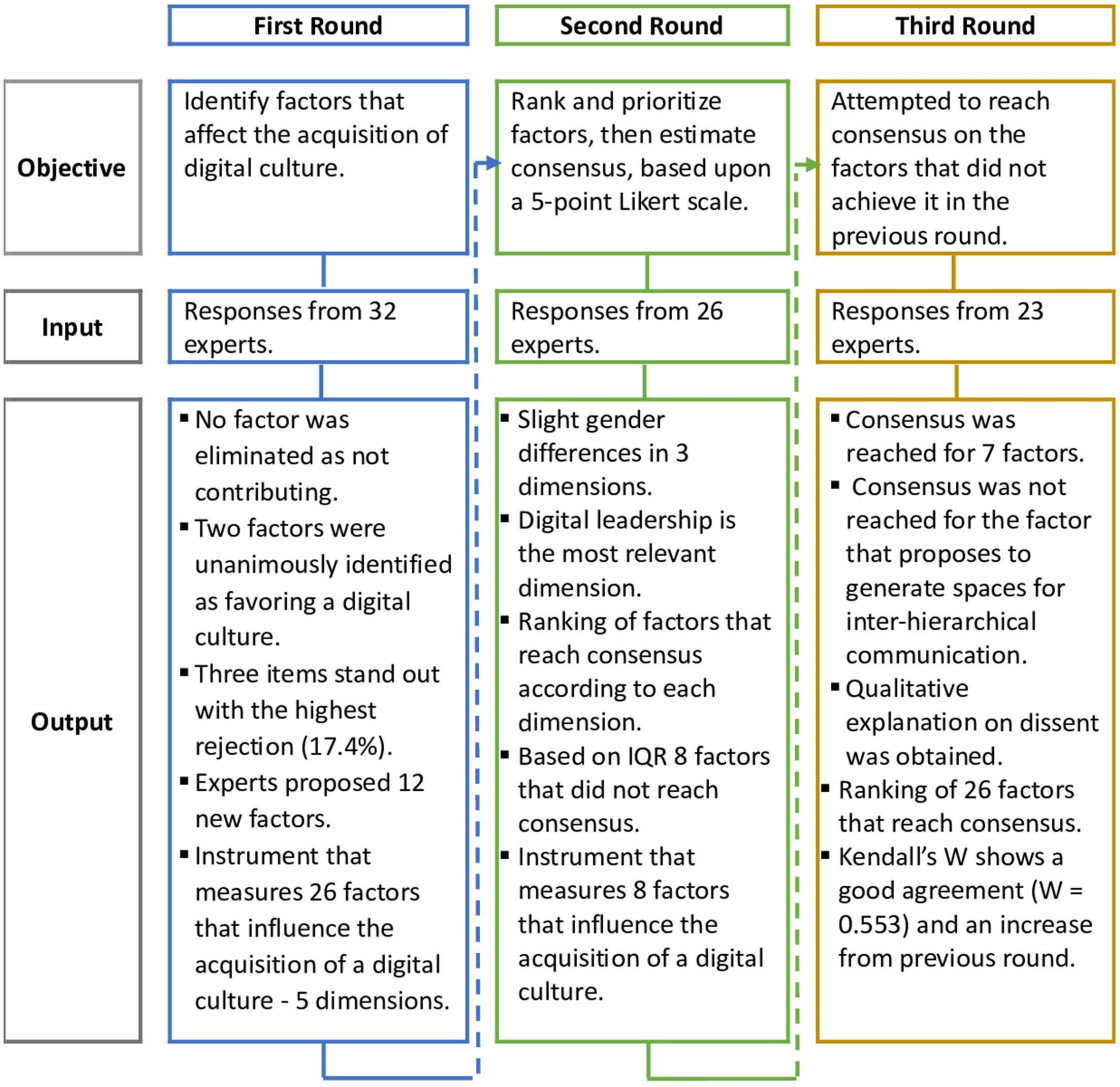
Objectives, inputs, and outputs for all three rounds.

A summary of the methodological objectives, inputs, and outputs during the three rounds is presented as a flow chart in Figure 2. Each round is rigorously analyzed in the following subsections.

### 4.1. First round

This first round intended to identify experts’ impressions on the factors that affect the acquisition of digital culture in large organizations in Chile. Data analysis identified 12 factors that had not been considered, and no factor was eliminated as not contributing to digital culture. Results can be seen in Table 2.

**Table 2:** Results from the first questionnaire.

Factors	Does not contribute	Difficulty’s the achievement	Favor’s the achievement
Continuously promote disruptive change	8.57%	17.14%	74.29%
Present a clear vision and goals for digital transformation	0	0	100%
Present a shared identity around the digital	17.14%	0	82.86%
Present a digital strategy aligned with the corporate strategy at a functional and operational level	0	0	100%
Motivate employees through incentives aligned with digital transformation	17.14%	0	82.86%
Encourage collaboration, risk-taking, and experimentation	11.43%	0	88.57%
Encourage risk-taking	14.29%	5.71%	80%
Foster the integration of cross-functional teams to implement digital initiatives	2.86%	2.86%	94.29%
Encourage cooperation between employees	14.29%	0	85.71%
Encourage effective communication.	17.14%	2.86%	80%
Develop an experimental and innovative culture	8.57%	0%	91.43%
Develop a collaborative culture	14.29%	0	85.71%
Develop a capacity for rapid adaptation	5.71%	2.86%	91.43%
Develop digital skills in senior management leaders	8.57%	0	91.43%

Two factors were unanimously identified as “favoring the achievement” of a digital culture: a clear vision and goals about digital transformation, and a digital strategy aligned with the corporate strategy at a functional and operational level. All other items were described by at least one expert as “not contributing to the achievement” of a digital culture and are considered in the next round because there is no consensus on the extent of their contribution. Three items stand out, with the highest rejection being 17.4%: present a shared identity around digital transformation, motivate employees through incentives that align with digital transformation, and encourage effective communication.

Five items were chosen from factors that consider how difficult the achievement of a digital culture is. Most of them have a very low percentage; however, continuously promote disruptive change stands out with a percentage of 17.14%.

Finally, the open question allowed participants to indicate factors that had not been mentioned previously and encouraged them to brainstorm about factors that had been overlooked. Among the most common responses were factors related to tolerance of failure, ways to properly manage change or learn from mistakes, leadership commitment to the digital culture project, training and digital education of employees, and orientation focused on customer needs, among others. These factors were included in the next round and made up for the 26 factors that influence the acquisition of a digital culture in large Chilean organizations, distributed in five dimensions (i.e., digital culture, digital leadership, digital strategy, digital skills, and technologies), which were previously presented and defined in Table 1.

### 4.2. Second round

The responses to this round allowed us to rank and prioritize factors and to establish the degree of dispersion of the responses. Although there is no single way to estimate consensus (Hallowell and Gambatese, 2010; Heiko, 2012), for this study, the main statistics are measures of central tendency and dispersion: media, maximum, minimum, interquartile range (IQR), and average range. In this case, media is the measure that best represents group opinion (Landeta, 1999), showing that digital leadership (5.0) is the most relevant dimension, followed by digital strategy (4.7). We can find digital culture (4.1), digital skills (4.0), and technology (4.0) with lower evaluations.

From a gender perspective, the media shows some differences in three dimensions: digital strategy, digital culture, and technology. For digital strategy, men indicate value with a media of 4.8, whereas women establish a media of 4.4. For the digital culture dimension, women were also stricter with an average of 4, whereas men averaged 4.3. Unlike the previous dimension, for technology, evaluations are frequently different for all factors. The main difference is regarding the item promote the use of digital tools within the company without the need for very advanced programming knowledge, where male experts average 4, whereas female experts average 3.5. Despite this variation between genders, all experts consider this item to be the least relevant category for digital culture success in large companies.

The maximum and the minimum indicate the extreme responses, whereas the IQR presents the location of the central half of the responses to measure the dispersion of the sample, which is inversely proportional to the group consensus (Climent et al., 2014). Based on this statistical analysis, we decided whether to keep or eliminate factors for the next round. If the item has an IQR that is less than or equal to 1, it is eliminated from the following questionnaire because consensus has been reached. We also estimated the average range, which is a more accurate measure than the average, because it allows us to distinguish factors with the same average and position them within a ranking. Based upon a 5-point Likert scale, the results of this round are presented in Table 3.

**Table 3:** Results for the second-round questionnaire.

Factors	Min.	Max.	Med.	IQR	Average range	Ranking	Consensus
**Digital culture**							
Encourage the generation of multifunctional/multidisciplinary teams to implement digital initiatives.	4	5	5	1	18.15	1	Yes
Raise awareness within the organization that error is a part of learning in the digital culture.	3	5	4.5	1	16.73	2	Yes
Generate a shared identity around digital within the organization.	3	5	4.5	1	16.40	3	Yes
Develop a capacity to adapt quickly to changes.	3	5	4	1	16.23	4	Yes
Promote proactivity, adaptability, and a quick and adequate response to changes in the environment	3	5	4	1	14.54	5	Yes
Develop a rapid and efficient response capacity to customers’ changing needs (business models, processes, etc.).	2	5	4.5	1.25	14.44	6	No
Encourage the organization to take risks.	3	5	4	1	14.25	7	Yes
Create working groups that unite the digital generation and the employees with experience to generate intergenerational support.	3	5	4	2	12.79	8	No
Continuously conduct low-cost experiments with digital technologies and/or prototypes.	1	5	4	2	12.29	9	No
Generate spaces for inter-hierarchical communication regarding digital transformation within companies.	2	5	4	2	10.04	10	No
Develop virtual spaces to work collaboratively (e.g., JIRA, Teamwork Project Management, Microsoft SharePoint, etc.).	1	5	4	1.25	9.81	11	No
Identify employee resistance to change and lack of participation and commitment to support the digital transformation of the company.	1	5	4	1.25	8.94	12	No
Continuously promote disruptive changes within the organization.	2	5	4	1.25	8.23	13	No
Create reward systems to strengthen the position and growth of highly qualified personnel (e.g., promotions, bonuses, awards, etc.)	1	5	3.5	1	4.94	14	Yes
**Digital leadership**							
Build a supportive culture that encompasses collaboration, risk taking, and experimentation through organizational leaders (senior management and/or directors).	3	5	5	1	18.10	1	Yes
Develop leadership (from top to bottom management) toward digital to use new technologies.	3	5	5	1	18.10	2	Yes
**Digital strategy**							
Present a digital strategy that aligns with the corporate strategy at a functional and operational level.	4	5	5	1	20.23	1	Yes
Define key results in KPI format.	3	5	5	1	17.67	2	Yes
Present a clear vision and objectives for digital transformation both in the medium and long term.	3	5	4.5	1	17.15	3	Yes
Properly manage change and communicate the expectations and predicted impacts to the employees.	3	5	4.5	1	15.77	4	Yes
Develop a change management area/team with a clear methodology within the company.	1	5	4	1	13.75	5	Yes
**Digital Skills**							
Invest in the comprehensive development of digital skills and abilities that align with the company’s strategy.	2	5	4	1	14.44	1	Yes
Permanently invest in digital training of employees at all levels of the company to develop up-to-date technological skills (e.g., training).	1	5	4	1	13.25	2	Yes
Selectively invest in the most talented, adaptable, curious and flexible people to develop, attract and retain the best talent in the field.	2	5	4	1	7.58	3	Yes
**Technological resources**							
Integrate digital initiatives (e.g., digitization of information, processes, etc.) throughout the company.	3	5	4	0.25	12.27	1	Yes
Promote the use of digital tools within the company without needing very advanced programming knowledge.	1	5	4	1.25	4.81	2	No

To complement the search for consensus that was determined by the IQR, we analyzed Kendall’s W (also known as Kendell’s coefficient of concordance). This non-parametric statistic is used to determine the degree of correlation between qualitative ordinal variables, which makes it possible to measure the agreement, the relative strength and the change in the experts’ responses (Heiko, 2012). Schmidt (1997) suggest the following interpretation (see Table 4).

**Table 4:** Kendall’s W (coefficient of concordance), based on Schmidt (1997).

W	Interpretation
W < 0.3	Weak agreement
0.3 < W < 0.5	Moderate agreement
0.5 < W < 0.7	Good agreement
W > 0.7	Strong agreement

For this round, Kendall’s W was measured to compare the degree of agreement obtained among factors that did or did not manage to reach consensus. As shown in Table 5, there is significant agreement between the scores assigned to the factors that reached consensus, evaluated as a good agreement (0.513). For the eight factors that did not reach consensus, Kendall’s W can be interpreted as a moderate agreement (0.424).

**Table 5:** Agreement index of factors with or without consensus in the 2nd round.

Statistics	Factors that reached agreement	Factors that did not reach agreement
Amount of experts	23	23
Kendall’s W	0.513	0.424
Significance level	<0.001	<0.001

### 4.3. Third round

In this last phase, according to the Delphi protocol, the experts attempted to reach a consensus on the factors that did not achieve agreement in the previous round. For the third questionnaire, consensus was reached for all factors, except for one that proposes to generate spaces for inter-hierarchical communication related to digital transformation within companies, which presented a greater dispersion in the scores and extremes of 2–5 points.

In this round, the factor that managed to position itself as the most relevant was develop a rapid and efficient response capacity to the changing needs of customers (Media = 5). Table 6 presents the results of this third questionnaire.

**Table 6:** Results from the third questionnaire.

Factors	Min.	Max.	Med.	IQR	Average range	Consensus
Develop the capacity to provide rapid and efficient responses to changing customer needs (e.g., business models, processes, etc.).	2	5	5	1	6.17	Yes
Generate spaces for inter-hierarchical communication regarding digital transformation within companies.	2	5	4	2	4.26	No
Develop virtual spaces to work collaboratively (e.g., JIRA, Teamwork Project Management, Microsoft SharePoint, etc.).	1	5	4	0	4.28	Yes
Continuously conduct low-cost experiments with digital technologies and/or prototypes.	1	5	4	1	5.33	Yes
Promote the use of digital tools within the company without needing very advanced programming knowledge.	1	5	4	1	2.72	Yes
Continuously promote disruptive changes within the organization.	2	5	4	1	3.74	Yes
Create working groups that unit the digital generation and the employees with experience to generate intergenerational support.	3	5	4	1	5.74	Yes
Identify employee resistance to change and lack of participation and commitment to support the company’s digital transformation.	1	5	4	1	3.76	Yes

Finally, the round of questionnaires concludes when the desired degree of stability and consensus has been achieved. The calculation of Kendall’s W considers all of the factors that reached an acceptable level of consensus and shows a significant agreement and an increase in the degree of agreement between participants, corresponding to a good agreement (W = 0.553). Table 7 shows the final ranking of all 26 factors that reached consensus.

**Table 7:** Ranking of factors that promote a digital culture in large companies in Chile.

Factors	Ranking
Align digital strategy with the corporate strategy at a functional and operational level.	1
Encourage the generation of multifunctional/multidisciplinary teams to implement digital initiatives.	2
Build a supportive culture that encompasses collaboration, risk taking, and experimentation through organizational leaders (senior management and/or directors).	3
Develop leadership (from top to bottom management) toward digital to use new technologies.	4
Define key results in KPI format.	5
Develop the capacity to provide rapid and efficient responses to changing customer needs (e.g., business models, processes, etc.).	6
Present a clear vision and objectives around digital transformation both in the medium and long term.	7
Raise awareness within the organization that errors are a part of learning in the digital culture.	8
Generate a shared identity around digital within the organization.	9
Properly manage change and communicate expectations and predicted impacts to the employees.	10
Develop a capacity to quickly adapt to changes.	11
Promote agility within the organization.	12
Invest in the comprehensive development of digital skills and abilities that align with the company’s strategy.	13
Encourage employees in creative areas (e.g., R&D, Marketing, HR, etc.) to take risks.	14
Develop a change management area/team with a clear methodology within the company.	15
Create working groups that unite the digital generation and the employees with experience to generate intergenerational support.	16
Permanently invest in digital training of employees at all levels of the company to develop up-to-date technological skills (e.g., training).	17
Integrate digital initiatives (e.g., digitization of information, processes, etc.) throughout the company.	18
Continuously conduct low-cost experiments with digital technologies and/or prototypes.	19
Generate spaces for inter-hierarchical communication regarding digital transformation within the company.	20
Develop virtual spaces to work collaboratively (e.g., JIRA, Teamwork Project Management, Microsoft SharePoint, etc.).	21
Identify employee resistance to change and lack of participation and commitment to support the company’s digital transformation.	22
Continuously promote disruptive changes within the organization.	23
Selectively invest in the most talented, adaptable, curious and flexible people to develop, attract and retain the best talent in the field.	24
Promote the use of digital tools within the company without needing very advanced programming knowledge.	25
Create reward systems to strengthen the position and growth of highly qualified personnel (e.g., promotions, bonuses, awards, etc.).	26

## 5. Discussion

The results can be divided in two categories: those that are aligned with the literature and those that are not. This represents a risk in achieving a digital culture in large Chilean companies.

According to high executives, digital leadership is the most relevant dimension to achieve a digital culture in large Chilean companies, followed by the digital strategy dimension. The medias show a high level of agreement among experts, and the IQR shows that there is little variability, indicating stability in the responses and consensus when ranking factors, regardless of gender differences.

One of the lessons learned from the COVID-19 pandemic and the acceleration of digital transformation in Chile (ITD, 2021) is that the entire management team must be involved in the digital culture process, including betting on people and technology, which is mentioned by some of the expert panelists. Nevertheless, the following attributes covered in this study must be fostered and enhanced in a comprehensive manner to achieve a real impact on employees: Share authority and power as leaders who are facilitators and motivational trainers, exchange information and knowledge, promote collaborative work to foster a digital environment where collaborators can develop their full potential and demonstrate all of their abilities, encourage creativity and innovation, and promote internal communication with the corresponding digital tools (Cortellazzo et al., 2019; Gabryelczyk, 2020).

Another result that is consistent with the literature is the idea among high executives that a digital culture can be reached when the digital transformation aligns with the company’s strategy. The results from the first questionnaire show that all of the experts consider this to be a favorable factor to achieve a digital culture in large Chilean organizations, which is confirmed in the second-round, positioning it as one of the most relevant factors. This is consistent with the literature that focuses on organizational change and the creation of flexibility to adapt to changing digital environments. However, creating an effective strategy and linking it to the overall goals of a business remains one of the biggest challenges preventing companies from increasing digital maturity (Kane et al., 2017).

The expert panel identified investing in digital skills as a relevant dimension in the acquisition of a digital culture. After the first-round, the participants suggested factors that are associated with digital learning to empower and help businesses overcome the challenges that arise with digital transformation. This dimension was added in the second-round and was evaluated as an important dimension for achieving a digital culture (Media = 4.0), in accordance with the literature (Ochoa, 2016; Villaplana and Stein, 2019).

The final observation relates to the relationship among organizational culture and its effects on digital maturity. According to participants, to acquire a digital culture, the following factors are relevant: encourage the generation of multifunctional/multidisciplinary teams to implement digital initiatives, exercise a culture of tolerance toward failure, develop a capacity to rapidly adapt to change, and encourage employees in creative areas to take risks.

Therefore, in accordance with the literature, digitally mature organizations accept failure as a natural part of experimenting with new initiatives, actively implement initiatives to increase agility in response to changing markets, value and encourage experimentation and testing as a means of organizational learning, recognize and reward collaboration between teams, acknowledge divisions as part of the operating model, and increasingly organize around cross-functional project teams to implement digital businesses (Chanias and Hess, 2016; Kane et al., 2017).

On the other hand, when analyzing the results, there are several details that do not match the literature, raising concerns on the aspects that hinder the leadership of Chilean high executives who aim to achieve digital cultures and to create digitally mature companies.

We found three factors that were described by some participants as not contributing to digital culture that, according to Teichert (2019), are among the most represented cultural attributes in all digital maturity models: encourage risk taking, encourage cooperation among employees, and develop a collaborative culture. The success of a digital culture is determined through collective work and information exchange between divisions, units, and roles, where collaboration is valued more than individual effort and where coordinating tasks effectively, integrating employees, and carrying out the mission, vision and values of the organization is not possible without effective organizational communication (Hemerling et al., 2018).

On the other hand, the literature highlights the relevance of implementing a reward and incentive system that aligns with the acquisition of a digital culture (Mirković et al., 2019), although the results from the first round of this study slightly contradict this matter. After the first round, 17.14% of experts considered creating a reward system to strengthen the position and growth of highly qualified personnel to be a factor that does not contribute to achieving a digital culture. In the second-round questionnaire, this item was valued as the least relevant for achieving such success (Media = 3.5), presenting a high level of agreement among the panel of experts that can be explained by Chileans hierarchical organizational cultures (Rodríguez, 2011).

Another misalignment between the representatives of large Chilean companies’ strategic apex and the international literature is the rejection (17.4% in the first-round) caused by continuously promoting disruptive changes within the organization. After the second round, this factor was ranked 23, near the end of the list of 26 factors. This result raises questions on the meaning that some companies in Chile attribute to the need to move away from stability. In companies, digital disruption is defined as an alteration produced by a shift due to the development of new technologies that completely changes business models and affects the entire corporate structure (Roblek et al., 2021). Therefore, digital transformation and disruptive technologies facilitate the creation of solutions, whereas agile working formats adapt to these constant changes (Lucas and Goh, 2009).

High executives often cling to the status quo and traditions, rather than being open and committed to adaptive change. The current volatile environment in Chile and around the world is influenced by economic, social, technological, and political changes and has generated a significant level of stress among high executives (ITD, 2021), who, after numerous decades, had become accustomed to a stable context where conservative and low-diversity countries such as Chile could succeed.

Chilean labor culture is characterized by gradual change over disruption, hierarchical leadership over collaboration, loyalty over performance, the company’s internal process over the client, betting on what works over innovation, focusing on the task over the purpose, and short-term results over value creation. For this kind of organizational culture, it is easier to understand digital transformation as an investment in new technologies rather than as a constant promotion of disruptive change, which creates a blind spot for achieving digital maturity (CLA-Icare, 2021).

Another example of this tendency toward a more conservative strategic approach is the factor that did not reach consensus and was left out of the final list of factors: generate spaces for inter-hierarchical communication regarding digital transformation within companies. This factor encompasses effective organizational communication within companies, both vertical and horizontal, that encourages interactions between the strategic apex and employees to increase productivity, knowledge exchange, and collaboration. However, a lack of effective internal communication demotivates employees and makes it difficult to attract talent.

## 6. Conclusion

The research question for this study was the following: What factors favor or hinder the acquisition of a digital culture in large Chilean organizations? The aim was to rank factors that promote a digital culture based on the perceptions of senior executives by using the Delphi method.

The contributions made by this research allow the management of big Chilean companies to become aware of several factors that are not considered as relevant as leadership and strategy, which hinders their ability to achieve a digital culture. In addition, these findings help senior executives of large companies promote a culture that fosters a successful digitalization process while recognizing the cultural factors that influence the success or failure of digital transformation initiatives. Furthermore, this study contributes to the literature by compiling the most relevant factors that any strategic leader should focus on when facing a digital transformation (see Table 3). Some research limitations regarding the applied methodology are mainly the number of rounds and number of participants. Both are related to limited resources and deadlines, and although the final number of participants conforms to Landeta’s (1999) recommendations for this method, additional experts may have suggested factors not found in this study. Likewise, as the rounds of questionnaires progress, the number of experts decreases, so there is data lost due to this lack of continuity.

On the other hand, this study was carried out with experts belonging to various industries, obtaining a general representation of the factors that affect the acquisition of a digital culture for large companies in Chile. Therefore, it is recommended that future research also consider and analyze the differences and similarities between industries, to gain a deeper understanding of the challenges of achieving an organizational digital culture when embracing the strategic objective of a digital transformation.

An interesting result comparison could be done using the same instruments but among large companies’ workers that are not part of the strategic apex. The relevance of leadership and strategy factors opposed to the “*relevance of generating spaces for inter-hierarchical communication*” may be explained by the sample’s position in the organizational structure, showing a significant blind spot among strategic decision makers. We also propose to develop the same research in large public organizations in Chile to compare results, identify weaknesses in the achievement of e-government and identify strengths and weaknesses to promote a digital labor culture as a country.

Given that organizational leaders are aware of most of the factors related to achieving a digital culture, future lines of research can focus on their achievement, evaluating implementation plans. Another line of research suggested is to focus on the slight gender differences observed. The relative lower evaluations made by women in the sample can raise new questions that can be answered by a more accurate statistical analysis and complemented by qualitative methodologies in search for deeper explanations.

In regard to digital cultures in large Chilean companies, the participants of the expert panel show a high level of agreement about the importance of factors that are primarily related to two dimensions: *digital strategy* and *digital leadership*.

Notably, the participants expressed concern about implementing technology through a workforce with digital skills, where the decision to replace the existing workforce should shift toward constant digital training to make this digital transformation sustainable.

However, large Chilean companies must pay attention to the conservative triad of factors that characterize Chilean work culture: the belief that changes are exclusively possible when commanded by the strategic apex of senior executives, a hierarchical work culture that prevents collaborative work, and the rejection of disruptive change. These factors and cultural characteristics of the majority of Chilean companies will most likely hinder any attempt to accomplish a digital transformation plan.

## Data availability statement

The raw data supporting the conclusions of this article will be made available by the authors, without undue reservation.

## Ethics statement

Ethical review and approval was not required for the study on human participants in accordance with the local legislation and institutional requirements. The patients/participants provided their written informed consent to participate in this study.

## Author contributions

CB and FG contributed to conception and design of the study and oversaw the methodological process and statistical analysis. CB wrote the first draft of the manuscript. MA contacted the sample and performed the methodology and the statistical analysis. All authors contributed to the article and approved the submitted version.

## Funding

Research funding for women professors provided by Universidad Diego Portales through the project “Indicators of digital maturity in the Chilean industry”, awarded in 2022. Open access funding provided by Universidad Diego Portales (UDP).

## Conflict of interest

The authors declare that the research was conducted in the absence of any commercial or financial relationships that could be construed as a potential conflict of interest.

## Publisher’s note

All claims expressed in this article are solely those of the authors and do not necessarily represent those of their affiliated organizations, or those of the publisher, the editors and the reviewers. Any product that may be evaluated in this article, or claim that may be made by its manufacturer, is not guaranteed or endorsed by the publisher.
